# Myocardial infarction during anaphylaxis in a young healthy male with normal coronary arteries- is epinephrine the culprit?

**DOI:** 10.1186/s12872-017-0670-7

**Published:** 2017-09-04

**Authors:** W. D. Jayamali, H. M. M. T. B. Herath, Aruna Kulathunga

**Affiliations:** National Hospital, Colombo, Sri Lanka

**Keywords:** Anaphylaxis, Epinephrine, Myocardial infarction

## Abstract

**Background:**

Anaphylaxis is an acute, potentially fatal medical emergency. Myocardial injury or infarction in the setting of an anaphylaxis can be due the anaphylaxis itself, when it is known as Kounis syndrome or it can also be due to the effect of epinephrine treatment. Epinephrine is considered as the cornerstone in management of anaphylaxis. Myocardial infarction secondary to therapeutic doses of adrenaline is a rare occurrence and only a few cases have been reported in literature. The mechanism of myocardial injury was considered to be due to coronary vasospasm secondary to epinephrine as the coronary angiograms were normal on these occasions.

**Case presentation:**

A 21-year- old previously healthy male got admitted to the local hospital with an urticarial rash and difficulty in breathing, one hour after ingestion of prawns for which he was known to be allergic. He was treated with 0.5 ml of intramuscular adrenaline (1:1000) which was administered to the lateral side of the thigh, following which he developed palpitations and tightening type central chest pain. Electrocardiogram showed ST segment depressions in leads III, aVF and V1 to V5 and he was transferred to a tertiary care hospital. The second electrocardiogram, done 2 h later, showed resolution of ST segment depressions but new T inversions in leads I and aVL. Troponin I was elevated with a titer of 2.15 ng/ml. He was treated with sublingual GTN in the emergency treatment unit and the symptoms resolved. Transthoracic 2D echocardiogram and stress testing with treadmill was normal and CT coronary angiogram revealed normal coronary arteries.

**Conclusion:**

Here we present a case of a young healthy adult with no significant risk factors for coronary artery disease who developed myocardial infarction following intramuscular administration of therapeutic dose of adrenalin for an anaphylactic reaction. The postulated mechanism is most likely an alpha receptor mediated coronary vascular spasm. However the use of adrenaline in the setting of life threatening anaphylaxis is life saving and the benefits far outweigh the risks of adverse effects. Therefore the purpose of reporting this case is not to discourage the use of adrenaline in anaphylaxis but to make aware of this potential adverse effect which can occur in the acute setting.

## Background

Myocardial infarction occurring in the context of an anaphylactic reaction can occur due two possible reasons. One being the anaphylaxis itself causing allergic myocardial infarction, known as Kounis syndrome and the other being the use of epinephrine resulting in myocardial injury. Epinephrine is considered as the cornerstone in the management of anaphylaxis. Intramuscular injection of 1:1000 adrenaline to a maximum dose of 0.5 mg is recommended in the management of anaphylaxis [1, 2]. Intravenous use is less often used in anaphylaxis. There have been a few cases of myocardial infarction occurring as a result of therapeutic doses adrenaline for anaphylaxis, reported in the previous case reports [3–7].The mechanism of myocardial injury was considered to be due to coronary vasospasm secondary to epinephrine as the coronary angiograms were normal in these occasions.

Here we report a case of a young healthy male who developed myocardial infarction following administration of therapeutic dose of intramuscular adrenaline for an anaphylaxis.

## Case presentation

**Fig. 1:**
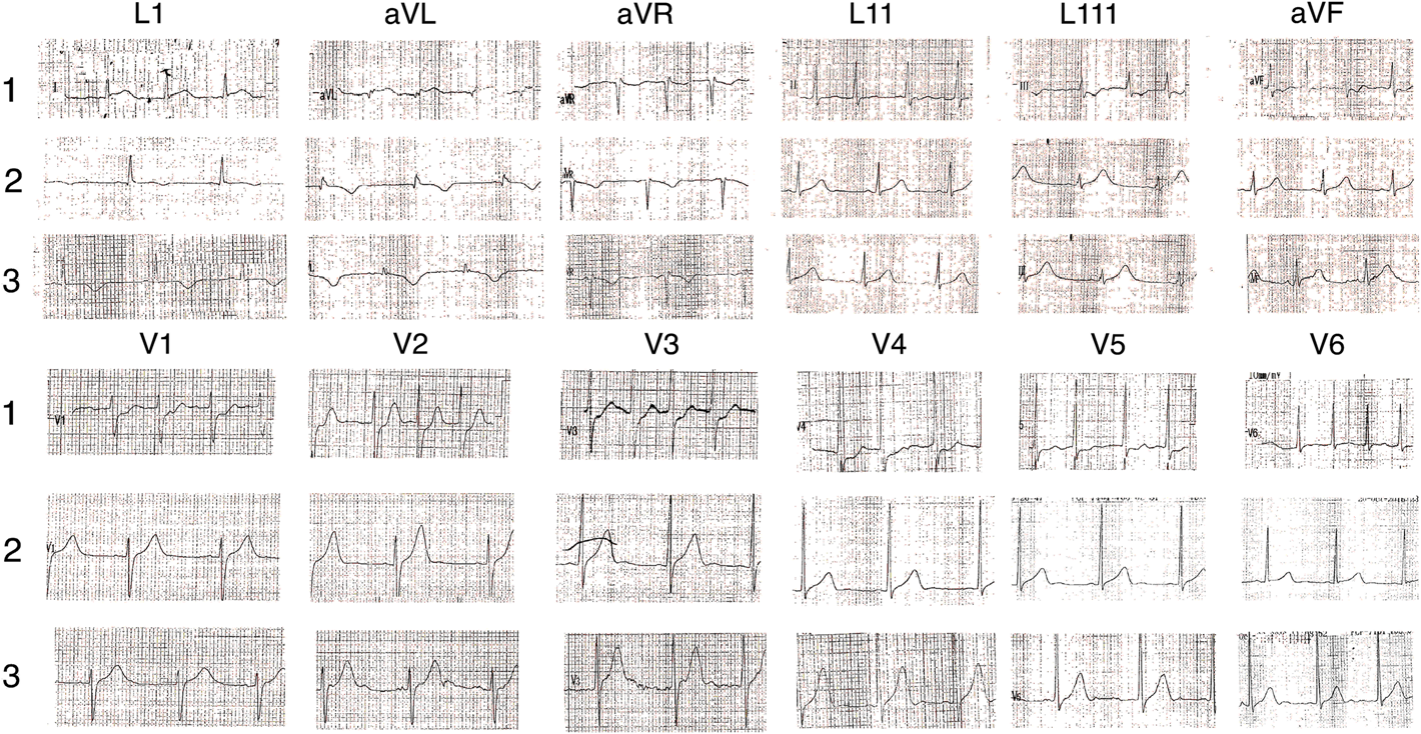
Electrocardiograms taken after adrenaline administration. 1 = The first ECG which taken at the local hospital (15 min after the chest pain / 25 min after the adrenalin) showing a sinus tachycardia of 109/min and ST segment depression in leads III, aVF and V1 to V5. 2 = The Second ECG taken 2 h after the 1st one showing resolution of ST segment depressions and new T inversions in leads I and aVL. 3 = T inversions persisted in subsequent ECGs

A 21-year-old Sri Lankan male developed urticaria and difficulty in breathing one hour after ingestion of prawns, for which he was known to be allergic. He got admitted to the local hospital 2 h after the onset of symptoms. On admission to the local hospital he was dyspnoeic with a respiratory rate of 28/min and widespread rhonchi. His pulse rate was 94 beats per minute and the blood pressure was 100/70 mmHg. He was treated with intravenous hydrocortisone 200 mg, intravenous chlorpheniramine 10 mg and 0.5 ml of adrenaline (1:1000 solution) intramuscularly to the upper lateral side of the thigh (vastus lateralis). Ten minutes after the administration of adrenalin, he developed palpitations and tightening type central chest pain with autonomic symptoms. The pain lasted for about 30 min and resolved spontaneously. The first electrocardiogram (ECG), which was taken at the local hospital showed a sinus tachycardia and ST segment depressions in leads III, aVF and V1 to V5(Fig. 1). He was not given any treatment for the chest pain in the local hospital and was transferred to our hospital about 2 h from the onset of the pain.

On admission to our hospital, he was not dyspnoeic and his pulse rate was 100 beats per minute and the blood pressure was 100/60 mmHg. His respiratory rate was 18/min and had a few rhonchi on auscultation. Rest of the examination was normal. The second ECG which was done in our hospital, 2 h after the 1st one, showed resolution of ST segment depressions but new T inversions in leads I and aVL (Fig. 1). These T in versions persisted in subsequent ECGs (Fig. 1). Troponin I done 6 h after the event was positive with a titer 2.15 ng/ml (<0.5). The test was repeated on the second day and it was still positive with a tire of 0.69 ng/ml. He was given sublingual glyceryl trinitrate 0.4 mg single dose after admission to our hospital. However antiplatelets and statins were not given and anticoagulation was not started as the most likely cause was assumed to be coronary vasospasm rather than plaque rupture.

He was previously healthy and did not have any risk factors for premature coronary vascular disease such as smoking. He has had a history urticaria to prawns but there was no previous history of anaphylaxis. He did not have asthma. There was no family history of diabetes, ischemic heart disease or premature deaths due to cardiovascular diseases. He worked as a computer operator trainee and was unmarried.

**Fig. 2:**
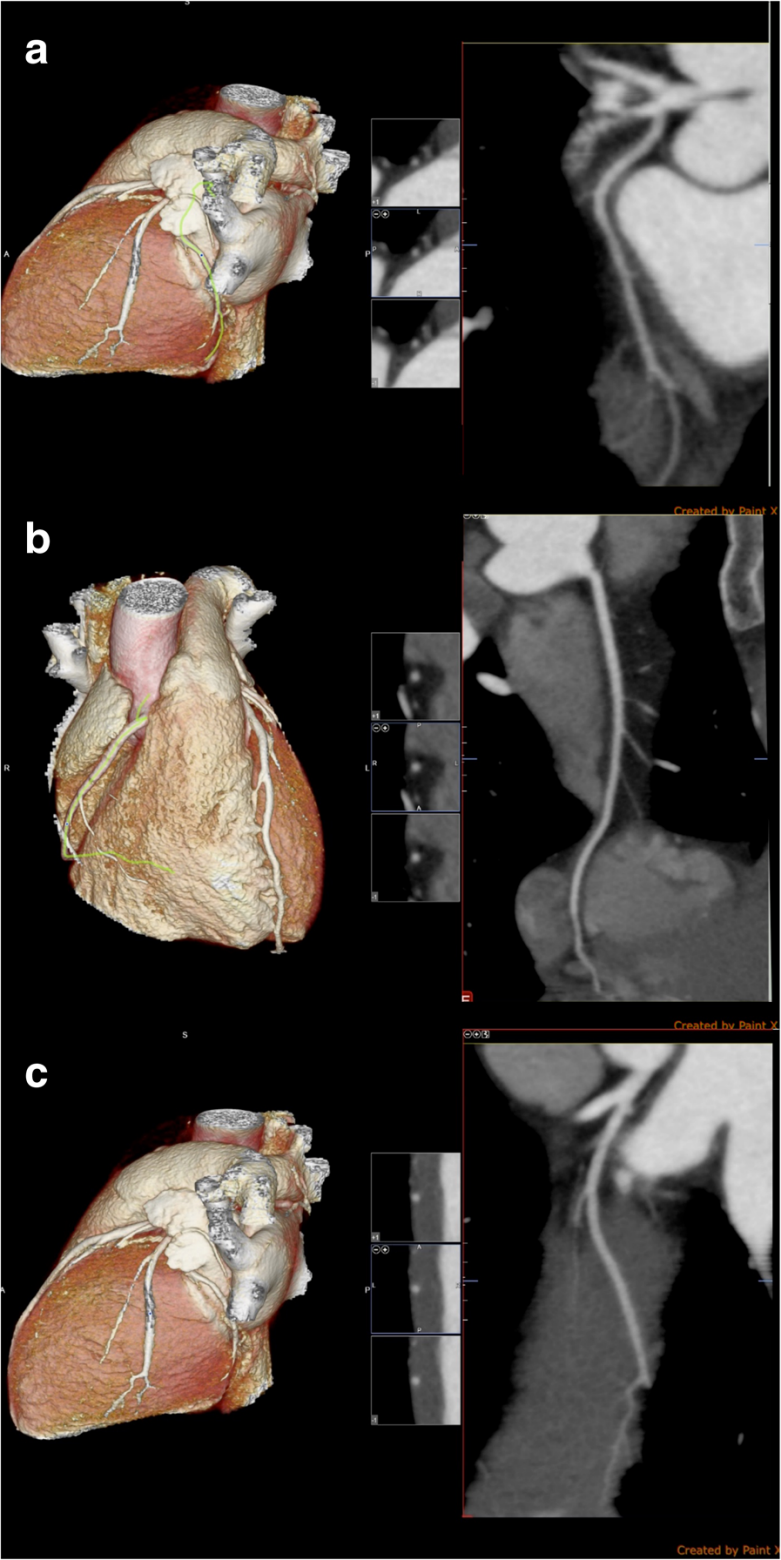
Normal CT coronary angiogram. **a** = Normal left circumflex artery. **b** = Normal Right coronary artery and posterior descending artery. **c** = Normal Ramus intermedius artery

Further investigations which were done at our unit included transthoracic 2D echocardiogram which revealed an ejection fraction of 60% with no wall motion abnormalities. We did not proceed with a coronary angiogram as the patient was a young healthy adult and the cardiology team concluded that coronary artery vasospasm to be the likely cause for the myocardial ischaemia rather than atherosclerotic coronary artery disease. Subsequent stress ECG with treadmill was normal and CT coronary angiogram revealed normal coronary arteries (Fig. 2). Complete blood count and renal functions were normal. Chest x ray was also normal. Fasting blood sugar was 98 mg/dl and the lipid profile was normal.

Patient was asymptomatic during the hospital stay and was discharged after 2 days. At subsequent reviews he remained asymptomatic.

## Discussion and conclusion

The patient presented in this case fulfilled the criteria for an acute myocardial infarction according to the universal definition of myocardial infarction as the patient had elevated cardiac troponins above 99th centile of the upper limit with a falling pattern with clinical evidence of ischemic type chest pain and significant new ST and T wave changes [8]. It is most likely type 2 myocardial infarction according to the third universal classification of myocardial infarction because the most likely underlying mechanism is coronary vasospasm [8].

Myocardial injury or infarction in the setting of an anaphylaxis can be due the anaphylaxis itself, when it is known as Kounis syndrome or can also due to the effect of epinephrine treatment. Kounis syndrome or allergic angina is an acute coronary syndrome occurring in association with conditions that involves mast cell activation such as hypersensitivity reaction,anaphylactic or anaphylactoid conditions [9–11].The chemical mediators that are involved are neutral proteases including tryptase and chymase, arachidonic acid products, histamine, platelet activating factor and a variety of cytokines and chemokines. It is believed that these mediators induce coronary vasospasm resulting in myocardial ischemia and infarction. Kounis syndrome consists of 3 variants. Type I is where patients with normal coronary arteries without predisposing factors for coronary artery disease develop coronary artery spasm with normal cardiac biomarkers or infarction with elevated biomarkers. This is most likely due to endothelial dysfunction or microvascular angina. The type II variant includes patients with inactive but quiescent preexisting atheromatous plaques, in whom the allergic insult leads to plaque erosion or rupture, leading to acute coronary event. The type III variant includes coronary artery stent thrombosis secondary to allergic reaction [9, 10]. There were several reported cases of patients where they have developed acute coronary syndrome following allergic reaction,most of which have occurred prior to receiving epinephrine and only one occasion where it occurred after the use of epinephrine [12, 13].

Epinephrine induced myocardial infarction in the setting of management of anaphylaxis has been reported only on a few occasions. There have been five reported cases where therapeutic doses of epinephrine had caused myocardial infarction [3–7]. Out of these reports one was after intravenous injection, another after intramuscular use of epinephrine and two cases following subcutaneous injection and one other where a subcutaneous epi pen auto injector use. The cases by Shaver and colleagues, Ferry and colleagues, Rubio and colleagues and Saff and colleagues were similar to our patient as the patients were previously healthy individuals and have developed myocardial infarction after use of epinephrine and normal coronary arteries were demonstrated later. However in the case reported by Cunnigton and colleagues, the patient described had been on propranolol before the incident and they have identified it as a potential predisposing factory for epinephrine induced vasospasm.

Our patient was a 21 year old young healthy male who did not have any modifiable or non-modifiable risk factors for coronary artery disease. He had minimal cardiovascular symptoms up to two hours from the onset of allergy, but developed chest pain and palpitations 10 min following administration of epinephrine with associated ECG changes and elevated cardiac biomarkers. This temporal relationship is in more favor of epinephrine as the cause of myocardial infarction rather than the Kounis syndrome, but still it cannot be excluded. A likely mechanism postulated for the myocardial infarction by epinephrine was coronary vasospasm induced by it as the subsequent CT coronary angiogram demonstrated normal coronary vasculature.

Epinephrine has a high affinity for beta1, beta 2, alpha 1 and alpha 2 receptors in cardiac and smooth muscles of the vascular walls [14]. Stimulation of Beta 1 and Beta 2 leads to increased cardiac contractility and rate and dilates coronary arteries whereas alpha 1 and 2 receptors mediate vasoconstriction including that of the coronary vasculature. At low doses, beta-adrenergic effects are predominant whereas at higher doses, alpha effects are more pronounced. [14]. Yasue and his colleagues did an experimental study on four patients with prinzmetal’s variant form of angina and demonstrated severe coronary artery spasm induced by administration of epinephrine and propranolol together [15].

Even though our patient developed myocardial infarction following intramuscular administration of epinephrine it was considered to be a relatively safe route of epinephrine administration [16]. From the limited data available regarding the safest route of administration of epinephrine in anaphylaxis it is demonstrated that intravenous route is associated with more cardiovascular events than the intramuscular route. This was shown in an observational study done by Campbell and his colleagues on 573 patients who presented with anaphylaxis and managed with epinephrine administered by various routes [17]. Risk of overdose of epinephrine in this group was higher with the intravenous route as well. Intravenous overdose of epinephrine was a known cause of coronary vasospasm [18]. However even local infiltration of therapeutic doses of epinephrine with the purpose of reducing bleeding is known to cause coronary vasospasm [19]. Patients who are at risk of developing coronary artery spasms following administration of epinephrine is an area that needs to be explored. Old age, preexisting coronary artery disease and being on a beta blocker were some of the risk factors for epinephrine induced myocardial ischemia based on available evidence [11, 20, 21].

However even though there are no randomized controlled trials available on the use of adrenaline in anaphylaxis it is clearly evident from the available literature that early use of adrenaline is life saving and associated with a better outcome than delayed use [22, 23]. Therefore even though myocardial ischemia could occur on rare occasions even with therapeutic doses of adrenalin, this should not prevent the early use of adrenaline as a lifesaving medication. There are no absolute contraindications for the use of adrenaline in the setting of life threatening anaphylaxis as the risk of anaphylaxis is far greater than the risk of adverse effects [24].The attending physician should be aware and be vigilant of the possible adverse effects of adrenaline and take necessary remediable steps if they occur.

Most occasions of myocardial injury secondary of adrenaline were treated with nitrates with intravenous infusions or sublingual administration and calcium channel blockers [3, 6]. Our patient was treated with sublingual nitrates in the emergency unit and was not started on further treatment as he remained asymptomatic.

Here we presented a case of a young healthy adult with no significant risk factors for coronary vascular disease who developed myocardial infarction following a therapeutic dose of intramuscular administration of adrenalin for an anaphylactic reaction. Even though intra muscular administration of adrenalin is considered a safe route, this case illustrates that on rare occasions myocardial ischemia could result. The postulated mechanism is most likely an alpha 1 receptor mediated coronary vascular spasm. However the use of adrenaline in the setting anaphylaxis is life saving and the benefits far outweighs the risks of adverse effects. Therefore the purpose of reporting this case is not to discourage the use of adrenaline in anaphylaxis but to make aware of this potential adverse effect which can occur in the acute setting.

## Abbreviations

ECG: Electrocardiogram
